# Assessment of preventive practices towards hepatitis B infection among nursing students in Bangladesh: role of knowledge, attitudes and sociodemographic factors

**DOI:** 10.1186/s12912-024-01870-8

**Published:** 2024-03-21

**Authors:** Sumaiya Sultana Tamanna, Kallol Deb Paul, Md. Hasan Al Banna, Zamia Zannat, Anup Kumar Paul, Sadia Sultana, Najim Z. Alshahrani, Sohan Talukder, Md. Nazmul Hassan

**Affiliations:** 1 Faculty of Nutrition and Food Science, Patuakhali Science and Technology University, Patuakhali, 8602 Bangladesh; 2 Department of Food Microbiology, Faculty of Nutrition and Food Science, Patuakhali Science and Technology University, Patuakhali, 8602 Bangladesh; 3 Nutrition Initiative, Kushtia, Bangladesh; 4 Department of Mathematics, University of Barishal, Barishal, 8254 Bangladesh; 5 Department of Family and Community Medicine, Faculty of Medicine, University of Jeddah, Jeddah, Saudi Arabia; 6 Department of Environmental Sanitation, Faculty of Nutrition and Food Science, Patuakhali Science and Technology University, Patuakhali, 8602 Bangladesh

**Keywords:** Hepatitis B, Knowledge, Attitude, Practice, Viral infection, Nursing students, Bangladesh

## Abstract

**Background:**

Globally, hepatitis B infection (HBI) poses a substantial public health concern and healthcare workers, including nursing students, are at a higher risk of contracting this disease. Thus, the study aimed to assess how knowledge, attitudes, and sociodemographic factors are associated with HBI prevention among a sample of Bangladeshi nursing students.

**Methods:**

A cross-sectional survey was performed among 737 nursing students from the nursing institutes of Khulna and Barishal divisions in Bangladesh from January to April 2023. The data were collected by providing questionnaires (structured questionnaire) in the classroom, following a stratified random sampling process. A model of multinomial logistic regression was used to evaluate the factors linked to HBI prevention practices.

**Result:**

The mean (SD) scores were 11.42 (± 2.88) for knowledge, 4.33 (± 1.91) for attitude and 4.27 (± 2.056) for practice respectively. Participants’ low knowledge (adjusted odds ratio, aOR = 2.562, 95% CI: 1.29–5.07) and poor attitude (aOR = 5.730, 95% CI: 3.19–10.28) regarding HBI were significantly associated with higher likelihood of poor practice towards HBI prevention. Moreover, being 2nd year of nursing students (aOR = 2.147, 95% CI: 1.19–3.86), being aged 19–20 years (aOR = 3.038, 95% CI: 1.30–7.09), being married (aOR = 0.320, 95%CI: 0.13–0.82) and having a family history of HBI (aOR = 0.134, 95%CI: 0.05–0.36) were significantly associated with poor practices of HBI prevention among study participants.

**Conclusion:**

The knowledge, attitude and practice scores of the nursing students on HBI prevention were suboptimal. We advocate for implementing regular HBI prevention education and policies, free or subsidized services, skill development, proper HBI prevention enforcement and strict professional ethics within nursing colleges. Such efforts should predominantly focus on second-year, aged 19–20 and unmarried nursing students.

**Supplementary Information:**

The online version contains supplementary material available at 10.1186/s12912-024-01870-8.

## Introduction

Contagious diseases in this modern era transcend geographical and political boundaries, thus posing a worldwide threat that jeopardizes all nations and individuals [1]. It is becoming a burning issue due to increasing morbidity and mortality [1]. Among the many contagious diseases present in developing countries, hepatitis B infection (HBI) is particularly widespread [2]. The fact that it is not well recognized by many people makes it even more dangerous because it can cause serious health effects, even death [3]. HBI (acute and chronic) of the liver that can lead to contagious liver disease, which is caused by the hepatitis B virus [4]. This chronic infection raises the possibility of difficulties such as liver failure, liver cirrhosis, cancer, and kidney disease [5, 6].

Viral hepatitis has a significant impact on people’s lives, societies, and healthcare systems [7]. Since 1990, mortality from viral hepatitis has climbed by 63%, making it the 7th leading cause of death globally [8]. It is extremely concerning because this is the only contagious disease whose death rates are increasing [8, 9]. In 2019, there were 296 million people affected by chronic hepatitis B, with 1.5 million new cases annually and it was accountable for 820,000 fatalities, the majority of which were due to hepatocellular carcinoma and cirrhosis (primary liver cancer) [5]. Now it is one of the world’s biggest public health issues because the hepatitis B virus (HBV) infects almost a third of people worldwide [10]. So, the third Sustainable Development Goal calls for the eradication of viral hepatitis, particularly HBI, by 2030 [11].

HBIs (chronic HBV) are comparatively common in the Southern part of Asia and Sub-Saharan Africa [4]. In Sub-Saharan Africa, the infection is reported to be 6.1%, with 87,890 deaths per year [5]. Several studies conducted in Ghana showed that the prevalence of HBI is higher than 10% among many research groups, including medical professionals [12]. Bangladesh, a country in South Asia, is in the HBV intermediate prevalence zone and it is a leading cause of illness, mainly chronic liver disease, and mortality [6]. It also responsible for 30% of acute hepatitis, 60% of liver cirrhosis, 75% of chronic hepatitis, and 65% of hepatocellular carcinoma in Bangladesh [6, 10]. A recent epidemiological study concluded that the general population of Bangladesh has an inadequate understanding of the control and management of HBI [13]. In the rural areas of Bangladesh, many people do not have appropriate knowledge about contagious disease (hepatitis B) [14]. They rely on different sources of knowledge and most of them are misconstrued, especially medical-related beliefs [15]. Consequently, Bangladesh ranks in the top ten countries in terms of viral hepatitis burden, with HBI accounting for the vast majority of cases, partly because of insufficient health education, illiteracy, poverty, and a lack of immunizations [16, 17]. From a public health perspective, an individual’s health-related behavior is influenced by his or her knowledge, attitudes, and practices (KAP) on a particular health condition [13, 18]. Given the limited healthcare resources in Bangladesh, assessing KAP towards hepatitis B in Bangladeshi people is crucial for reducing disease burden and developing policy schemes.

Several studies have been undertaken in Bangladesh to address this topic, particularly among the general population, students, patients and their attendants, nurses, and barbers [13, 19–22]. However, no studies have been conducted on KAP for hepatitis B among nursing students in Bangladesh. Nursing students are vulnerable to this infection (hepatitis B) as they are trainees of medical professionals and future healthcare workers. Since HBI is a public health issue and an occupational hazard for healthcare workers [11], nursing students must have a proper understanding of its preventive measures to protect themselves and raise awareness in the community. Moreover, the KAP for hepatitis B of nursing students can highlight the pre-requisite actions that should be implemented to avoid or minimize the transmission risk among healthcare workers as well as provide an approximate evaluation of non-medical people’s understanding [23]. To bridge the research gap, we aimed to assess how knowledge, attitudes, and socio-demographic factors are associated with preventive practices for HBI among a sample of nursing students in Bangladesh. We hypothesized that low knowledge, poor attitudes and socio-demographic factors would be associated with poor practices of HBI prevention among study participants.

## Methods

### Study area, participants, and sampling technique

This study was carried out among nursing students of the Barishal and Khulna divisions, specifically Khulna, Barishal, Jashore and Patuakhali districts in Bangladesh. These two divisions were purposefully chosen since, in Bangladesh, Barishal ranked second and Khulna ranked third, with 27% and 23% of cases of acute HBI, respectively [24]. Additionally, the incidences of nonalcoholic fatty liver disease and hepatic encephalopathy among chronic liver disease were also high [24]. Data collectors visited randomly selected nursing colleges. Participants were selected using a stratified random sampling technique. Stratification was performed considering various factors such as nursing program, year of study, and gender. The data was collected from the participants in their classroom during the break of their classes.

### Inclusion and exclusion criteria

All the participants were in the diploma and midwifery nursing program. Male and female students were included within the age range between 18 and 26 because that is the satisfactory age range for nursing students. Second and final year students were chosen as participants for this research. The 1st year students were excluded from this study because of their less interaction with the patients. Moreover, nursing students undergoing internships were excluded due to their inaccessibility.

### Sample size calculation

The Cochran formula was used to calculate the sample size [25]. The sample size was estimated based on the following parameters: (i) we used 50% as a predictive prevalence (i.e., *p* = 0.5) due to the lack of data on preventive practices towards hepatitis B among nursing students in Bangladesh, (ii) 95% confidence level (Z = 1.96), and (iii) 5% margin of error (e = 0.05).

Thus, minimum sample size for this study was, $n=\frac{{z}^{2}pq}{{d}^{2}}= \frac{{\left(1.960\right)}^{2} \left(0.5\right) (1-0.5)}{{\left(0.05\right)}^{2}}$ = 384.16 ≈ 384

Using a 20% non-response rate, the optimum sample size was 461. Instead of 461 samples, we took 737 because larger samples better reflect the population and provide more accuracy.

### Data collection tools, procedure, and quality control

A cross-sectional study was conducted between January and April 2023 among students at nursing colleges. A structured questionnaire was adopted from a previous study and modified to make it appropriate for our demographic [23]. For instance, we changed the demographic questions based on our country’s perspective (e.g., family income categories, academic year, etc.) and translated it to our native language for better understanding. Additionally, we changed some question categories to make it convenient for the students. During pilot testing, we asked the students about their understanding of the questionnaire and noted their feedback. Based on their feedback, we made some modifications without changing the actual meaning of the questions. The questionnaire was converted into Bengali from English and then back translated into English from Bengali by a translator to ensure that the questionnaire’s uniformity was maintained. An independent research assistant double-checked the procedure. Prior to data collection, a pilot test was done among 5% of our sample size. Uncertain questions were modified based on the results of the pilot testing. These 5% of participants were excluded in our main study population. A questionnaire-based data collection method was used to collect information. Prior to data collection, everyone was asked if he/she wanted to participate or not. We only collected data from those who willingly participated. If anyone said that they did not want to participate, we thanked them and went forward. Information was provided about the study’s aims and verbal consent was obtained from the participants. During the interview, individuals were also informed that their participation in this survey was entirely voluntary and they were under no obligation to continue.

### Variable and measure

The survey questionnaire consisted of a total of 43 questions, which included 9 demographic variables (Part A), 18 questions to determine knowledge (Part B), 8 attitude-related questions to determine attitude (Part C), and 8 questions related to practice to determine practice (Part D). All participants were required to answer all the questions from Part A to Part D.

The knowledge questions contained three options: Yes, No, and Not sure. The scoring was 1 for each correct answer and 0 for each wrong answer. The total score for knowledge was 18 and was categorized as low (0–9), moderate (10–14), and high (15–18). The attitude section was coded on five-point Likert scale, with responses of “strongly agree,” “agree,” “uncertain,” “disagree,” and “strongly disagree.” The scoring for the attitude section was done in the same way as the knowledge section, with 1 for each correct answer and 0 for each wrong answer. The total score for attitudes was 8 and was categorized as poor (0–4), moderate (5–6), and good (7–8). Practice scoring and categories were the same as attitude [23].

### Data analysis

The quantitative data was analyzed using SPSS for Windows Version 25.0. Descriptive statistics, such as frequency, percentages, mean, median and standard deviation were used to analyze the demographic details of the students. Chi-square test was done to see significant difference among the distribution. Logistic regression was performed to estimate the relationship between a dependent variable and one or more independent variable. Multicollinearity was checked before establishing logistic regression. In multinomial logistic regression analysis, the significant variables and those with p-value < 0.25 in the chi-square function were entered into adjusted regression model. The reliability test of the instrument was performed and the Cronbach’s alpha of knowledge was 0.712, attitude was 0.653 and practice was 0.678.

## Results

### Socio-demographic information

Of the total 737 participants, the majority were female (88.3%). The participants were between 18 and 24 years old. Two-thirds of participants (65.4%) were studying in the third year of their study. The majority of the respondents (95.5%) had no family history of hepatitis B. Table 1 presents participants’ socio-demographic data.

**Table 1:** Socio-demographic information of study participants (*n* = 737)

Variables	Categories	Frequency (%)
Age	19–20	117 (15.9)
	21–22	445 (60.4)
	23–24	175 (23.7)
Gender	Male	86 (11.7)
	Female	651 (88.3)
Religion	Hinduism	254 (34.5)
	Muslim	483 (65.5)
Marital Status	Married	28 (3.8)
	Unmarried	709 (96.2)
Academic Status	2nd year	255 (34.6)
	3rd year	482 (65.4)
Monthly Family Income	≤ 20,000 BDT	509 (69.1)
	21,000–40,000 BDT	176 (23.9)
	> 40,000 BDT	52 (7.1)
Educational Qualification of Father	Not Educated or primary level	189 (25.6)
	Secondary or Higher Secondary level	441 (59.8)
	Graduated or above	107 (14.5)
Educational Qualification of Mother	Not Educated or primary level	229 (31.1)
	Secondary or Higher Secondary level	470 (63.8)
	Graduated or above	38 (5.2)
Family History with HB Infection	Yes	33 (4.5)
	No	704 (95.5)

### Knowledge, attitude, and practice of preventing hepatitis B infection

The overview of knowledge levels about HBI prevention among nursing students are highlighted in Table 2. The majority of the participants (90.5%) knew that the dsDNA virus is responsible for HBI. The study participants showed varying levels of knowledge about the clinical manifestations of HBI, with 74.6% identifying jaundice as a symptom, more than a half (55.1%) acknowledging that not all infected individuals display symptoms and three quarters (75.6%) acknowledging the infectiousness of carriers (Table 2).

**Table 2:** Knowledge of hepatitis B infection prevention among study participants (*n* = 737)

Variables	Frequency (%)		
	Yes	No	Not Sure
A partial dsDNA (double-stranded DNA) virus causes hepatitis B infection.	667 (90.5)	40 (5.4)	30 (4.1)
Jaundice is a symptom of HB infection.	550 (74.6)	134 (18.2)	53 (7.2)
Not all hepatitis B infected people show signs/symptoms.	406 (55.1)	218 (29.6)	113 (15.3)
Carriers of hepatitis B (not sick) can pass the infection to others.	557 (75.6)	122 (16.6)	58 (7.9)
Hepatitis B virus do not transmit via the faeco-oral route.	250 (33.9)	268 (36.4)	219 (29.7)
Hepatitis B virus not transmitted through casual contact.	595 (80.7)	66 (9.0)	76 (10.3)
Hepatitis B infection can be transmitted through contaminated blood and blood products.	572 (77.6)	64 (8.7)	101 (13.7)
Hepatitis B infection can be transmitted by needles, unsterilized syringes, and surgical instruments.	654 (88.7)	46 (6.2)	37 (5.0)
Hepatitis B infection can be transmitted via unprotected sex.	603 (81.8)	72 (9.8)	62 (8.4)
Hepatitis B infection can be passed from a mother to her baby at birth.	621 (84.3)	56 (7.6)	60 (8.1)
Hepatitis B infection can be diagnosed by serological Rapid Diagnostic Test.	276 (37.4)	74 (10.0)	387 (52.5)
Diagnosis of HB infection can be done by a molecular test.	294 (39.9)	115 (15.6)	328 (44.5)
Hepatitis B infection is incurable.	241 (32.7)	386 (52.4)	110 (14.9)
Hepatitis B virus has the potential to cause liver cancer.	469 (63.6)	89 (12.1)	179 (24.3)
Hepatitis B vaccine is not made from human blood.	309 (41.9)	79 (10.7)	349 (47.4)
Hepatitis B vaccination prevent Hepatitis B infection.	609 (82.6)	38 (5.2)	90 (12.2)
Hepatitis B vaccine protect against liver cancer.	380 (51.6)	99 (13.4)	258 (35.0)
Hepatitis B infection have post-exposure prophylaxis.	361 (49.0)	75 (10.2)	301 (40.8)
Yes = Correct response; No and Not Sure = Wrong Response.			

The summary of responses of attitudes towards HBI prevention is presented in Table 3. The majority of respondents (87.4%) believed that they were not at risk of getting a HBI, but 53.9% of them recognized that any blood exposure carries inherent risks (Table 3).

**Table 3:** Attitude towards hepatitis B infection prevention (*n* = 737)

Variables	Frequency (%)	
	Yes	No
You are not at risk of getting hepatitis B infection	644 (87.4)	93 (12.6)
Blood exposure occasionally will not necessarily elevate the scope of contracting an HB infection.	340 (46.1)	397 (53.9)
Wearing personal protective equipment during surgery is unnecessary	196 (26.6)	541 (73.4)
Hepatitis B infection is not as serious as HIV infection	125 (17.0)	612 (83.0)
Hepatitis B infection is not serious since those who acquire it live normal lives.	267 (36.2)	470 (63.8)
Hepatitis B infection is not potentially serious because it is treatable	407 (55.2)	330 (44.8)
Needle pricks don’t require reporting	343 (46.5)	394 (53.5)
Blood or body fluid splashes on the face don’t require reporting	381 (51.7)	356 (48.3)

Note: Yes = Wrong response; No = Correct response

Table 4 illustrates the practices taken by the nursing students to prevent HBI. The frequency of the practices was reported as; hepatitis B screening (65.4%), hepatitis B vaccination (68.4%), vaccination ≥ 3 doses (52.6%), post-hepatitis B vaccination antibody testing (8.7%), changed gloves per client (67.6%), never recapped needles (28.4%), never splashed blood on the body (52.9%) and sometimes needle stick injuries in the past (50.6%).

**Table 4:** Practice of hepatitis B infection prevention among study participants (*n* = 737)

Variables	Categories	Frequency (%)
Screening for Hepatitis B infection	Yes	482 (65.4)
	No	255 (34.6)
Hepatitis B vaccination	Yes	504 (68.4)
	No	233 (31.6)
Number of doses	< 3 doses	349 (47.4)
	≥ 3 doses	388 (52.6)
Post hepatitis B vaccination antibody test	Yes	64 (8.7)
	No	440 (59.7)
	Not Applicable	233 (31.6)
Changing gloves for each patient, during blood collection	Always	498 (67.6)
	Sometimes	193 (26.2)
	Never	46 (6.2)
Recapping needles after use	Always	475 (64.5)
	Sometimes	53 (7.2)
	Never	209 (28.4)
Needle stick injuries in the past	Always	15 (2.0)
	Sometimes	373 (50.6)
	Never	349 (47.4)
Splashed blood/body fluids on body	Always	6 (0.8)
	Sometimes	341 (46.3)
	Never	390 (52.9)

### Overall KAP scores for the prevention of hepatitis B infection

The mean (SD) knowledge score among study participants was 11.42 (± 2.88) on a scale of 17.00. The mean (SD) attitude score was 4.33 (± 1.91) out of 8.00. The mean practice score for preventing HBI was 4.27 (± 2.056) out of 8.00.

**Fig. 1:**
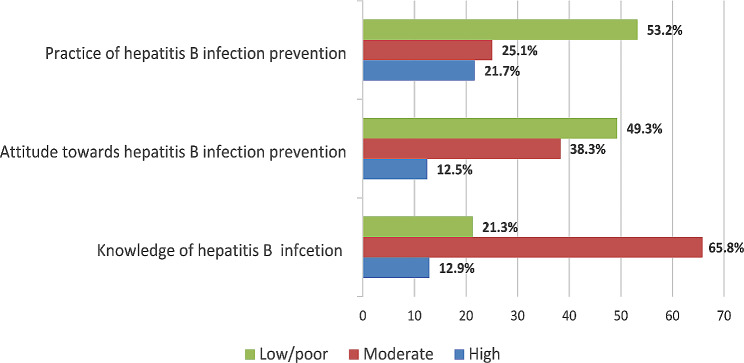
Level of knowledge, attitude and practice of hepatitis B infection among study participants (*n* = 737)

Overall, two-thirds of the respondents (65.8%) knowledge was moderate, and one-fifth (21.3%) was low. Nearly half of the respondents (49.3%) had poor attitudes toward HBI prevention. 53.2% participants had poor practice of HBI prevention and quarter of participants (25.1%) had moderate practices (Fig. 1).

### Factors associated with preventive practices of hepatitis B infection

The chi-square test shows that participants’ marital status (chi-square, X^2^ = 11.176, *p* = 0.004), family history with HBI (X^2^ = 23.809, *p* = 0.000), knowledge (X^2^ = 33.702, *p* = 0.000) and attitude (X^2^ = 63.630, *p* = 0.000) were significantly correlated with the HBI prevention practice (Table 5). In addition, the findings of unadjusted multinomial regression analysis are shown in the Supplementary Table 1.

**Table 5:** Distribution of variables among nursing students by practice of hepatitis B infection prevention

Variables		Practice of hepatitis B infection prevention							
		Good	(%)	Moderate	(%)	Poor	(%)	X^2^	*p*- value
Age	19–20	22	18.8%	21	17.9%	74	63.2%	7.833	0.098
	21–22	105	23.6%	226	25.6%	226	50.8%		
	23–24	33	18.9%	92	28.6%	92	52.6%		
Gender	Male	16	18.6%	21	24.4%	49	57.0%	0.713	0.700
	Female	144	22.1%	164	25.2%	343	52.7%		
Religion	Hinduism	48	18.9%	64	25.2%	142	55.9%	1.951	0.377
	Muslim	112	23.2%	121	25.1%	250	51.8%		
Marital Status	Married	13	46.4%	3	10.7%	12	42.9%	11.176	0.004
	Unmarried	147	20.7%	182	25.7%	380	53.6%		
Academic Status	2nd year	46	18.0%	60	23.5%	149	58.4%	4.819	0.090
	3rd year	114	23.7%	125	25.9%	243	50.4%		
Monthly Family Income (BDT)	≤ 20,000	108	21.2%	127	25.0%	274	53.8%	0.334	0.988
	21,000–40,000	40	22.7%	45	25.6%	91	51.7%		
	> 40,000	12	23.1%	13	25.0%	27	51.9%		
Educational Qualification of Father	Not Educated or primary level	39	20.6%	52	27.5%	98	51.9%	1.973	0.741
	Secondary or Higher Secondary level	100	22.7%	103	23.4%	238	54.0%		
	Graduated or above	21	19.6%	30	28.0%	56	52.3%		
Educational Qualification of Mother	Not Educated or primary level	47	20.5%	53	23.1%	129	56.3%	4.829	0.305
	Secondary or Higher Secondary level	100	21.3%	124	26.4%	246	52.3%		
	Graduated or above	13	34.2%	8	21.1%	17	44.7%		
Family History with HB Infection	Yes	18	54.5%	8	24.2%	7	21.2%	23.809	0.000
	No	142	20.2%	177	25.1%	385	54.7%		
Knowledge	High	40	42.1%	22	23.2%	33	34.7%	33.702	0.000
	Moderate	86	17.7%	115	23.7%	284	58.6%		
	Low	34	21.7%	48	30.6%	75	47.8%		
Attitude	Good	49	53.3%	16	17.4%	27	29.3%	63.630	0.000
	Moderate	46	16.3%	81	28.7%	155	55.0%		
	Poor	65	17.9%	88	24.2%	210	57.9%		

Table 6 represents the results of adjusted estimated effects concerning factors related to hepatitis B prevention practice. Nursing students with low knowledge regarding HBI prevention were found to be approximately three times more likely to engage in poor prevention practices when compared to those with a higher level of knowledge (aOR = 2.562; 95% CI = 1.29–5.07; *p* = 0.007). Nursing students who had poor attitudes towards preventing HBI were approximately seven times more likely to demonstrate poor HBI prevention practice in contrast to those with good attitudes (aOR = 5.730; 95% CI = 3.19–10.28; *p* = 0.000). Also, in contrast to unmarried students, married students were found to have a reduced likelihood of exhibiting poor practices for preventing HBI (aOR = 0.320; 95% CI = 0.13–0.82; *p* = 0.017). Nursing students in the 19–20 age group exhibited a threefold higher likelihood of showing poor practices for HBI prevention compared to those in the 23–24 age group (aOR = 3.038; 95% CI = 1.30–7.09; *p* = 0.010). In contrast to 3rd year students, 2nd year students were about twofold more likely to execute poor practices of HBI prevention than good practices (aOR = 2.147; 95% CI = 1.19–3.86; *p* = 0.011). However, nursing students with a family history of HBI were less likely to practice poor HBI prevention than those without one (aOR = 0.134; 95% CI = 0.05–0.36; *p* = 0.000) in contrast to those without.

**Table 6:** Multinomial logistic regression analysis for determining factors affecting practice of hepatitis B infection prevention (*n* = 737)

Variables		Practice of hepatitis B infection prevention					
		Moderate			Poor		
		aOR	95% CI	*p*-value	aOR	95% CI	*p*-value
Knowledge	High	Ref.			Ref.		
	Moderate	2.576	1.39–4.78	**0.003**	4.495	2.56–7.88	**0.000**
	Low	2.642	1.27–5.52	**0.010**	2.562	1.29–5.07	**0.007**
Attitude	Good	Ref.			Ref.		
	Moderate	5.009	2.53–9.94	**0.000**	5.834	3.19–10.68	**0.000**
	Poor	3.999	2.05–7.82	**0.000**	5.730	3.19–10.28	**0.000**
Age	19–20	0.776	0.29–2.03	0.606	3.038	1.30–7.09	**0.010**
	21–22	0.755	0.37–1.54	0.440	1.406	0.74–2.68	0.299
	23–24	Ref.			Ref.		
Marital Status	Married	0.179	0.05–0.68	**0.012**	0.320	0.13–0.82	**0.017**
	Unmarried	Ref.			Ref.		
Academic Status	2nd year	0.952	0.49–1.85	0.885	2.147	1.19–3.86	**0.011**
	3rd year	Ref.			Ref.		
Family History with HB Infection	Yes	0.409	0.16–1.02	0.056	0.134	0.05–0.36	**0.000**
	No	Ref.			Ref.		

Note: Bolded values indicate statistically significant (*p* < 0.05).

## Discussion

This study represents the first known research conducted in Bangladesh that demonstrates how nursing students’ knowledge, attitude and sociodemographic factors influence the prevention practice related to HBI. Overall, the study participants had moderate prevention knowledge of HBI. Several studies carried out in Ghana also demonstrate moderate knowledge, which is equivalent to our study [23, 26, 27]. In contrast to the current study, Hang Pham et al. in Vietnam found the poor knowledge of healthcare workers on the prevention of HBI [28]. The dissimilarity may be attributed to the smaller sample size of this study compared to ours. Our study outcome implies that the education on HBI prevention provided to these students was non-optimal. The poor knowledge led to an unfavorable environment and incapability, which made the nursing students unable to manage their own and other people’s health and that hindered effective disease prevention practice of HB. Therefore, empowering and motivating nursing students with consistent education is crucial for infection prevention.

The results of the current survey indicate a poor attitude towards HBI prevention among the study participants. Almualm et al. in Yemen also found that the vast majority of participants had a poor attitude about hepatitis B, which is parallel to our findings [29]. The findings differed from the study of Akazong et al. [30] who found a moderate level of attitude and Mursy and Mohamed et al. [18] who reported a good level of attitude about this infection prevention. The different outcomes observed across different studies may be attributed to the difference in assessing the attitude of working nurses in a hospital setting who possess work experience, in contrast to our study, which focuses on assessing the attitude of nursing students who are still in the process of studying and do not have any work experience. Balegha et al. suggest that the discrepancies in findings among different studies may be linked to the students’ differing levels of perception regarding the severity, risk and threat posed by HBI [23].

The findings of the present study disclosed that the practice of HBI prevention was poor. Balegha et al. [23], Afihene et al. [31], and Zaeri et al. [32] reported poor prevention practice for HBI. The findings are consistent to our study, as per their respective research studies. A significant proportion of the participants demonstrated a lack of practice regarding safety measures. The poor practice is caused by inadequate knowledge and awareness of HBI prevention [33]. However, the findings differed from the study by Mursy and Mohamed [18] in Sudan, which found safe practice towards HBI prevention. The observed dissimilarities can be attributed to variations in the respondents’ level of knowledge and attitude, as well as the principles of social cognitive theory [34].

There was a significant statistical correlation between having low knowledge about preventing HBI and an increased likelihood of engaging in poor practices to prevent HBI. This is coherent with previous studies in Ghana [27] and the Kingdom of Saudi Arabia [32].The correlation arises due to the adoption of the same sampling technique and analytical approach in both studies. This implies that acquiring insufficient knowledge impairs one’s health awareness [35], which can impede the adoption of preventive measures against hepatitis B, such as needle recapping after use to avoid exposure, among other activities. Low knowledge of HBI prevention hampers their ability to acquire and maintain the necessary skills that empower them to practice in the practical field.

Poor attitudes toward HBI prevention was highly correlated with a higher likelihood of poor practice. Similar findings were made by Ul-Haq et al. [36], Afihene et al. [31], and Balegha et al. [23] in Pakistan and Ghana. All the studies indicated that the participants exhibited poor attitudes, which were strongly associated with their inadequate practices regarding hepatitis B infection. Consistent with these findings, our study also identified a poor attitude among participants, thereby establishing a similarity between our study and the aforementioned research. The health belief model suggests that a negative attitude towards hepatitis B prevention, derived from the perception of seriousness, vulnerability, and the level of threat, can directly influence nursing students’ adherence to poor practices [37] Additionally, attitude and knowledge can interact to influence HBI practices [23]. Therefore, to promote the nursing student’s health, it is crucial to encourage them to adopt positive attitudes that help to maintain good practices towards individual and community health promotion.

The findings of this study revealed that participants in the 19 to 20 (younger) age group demonstrate a higher likelihood of poor practice in the prevention of HBI in contrast to those in the 23 to 24 (elderly) age group. A study conducted in Ethiopia [38] refutes our finding; they found that elderly students were more likely to have poor practice than younger students. These discrepancies are most likely due to the variations in the focus on different target group. However, one possible link could be that their knowledge grew with education [37].The elderly are at a greater risk of being exposed to HBI compared to younger ones [39], which may make them more sincere about hepatitis B prevention practice.

This study found that married participants had a reduced likelihood of poor practices toward HBI prevention as compared to unmarried participants, which is analogous to a previous study conducted in Malaysia [40] but the results obtained in Nepal [41] and Lagos State in Nigeria [42] were not congruent. Marriage fosters a feeling of responsibility and health consciousness [43], leading to the prioritization of health and disease prevention [40], including hepatitis B prevention. However, further research is required to properly investigate the exact role of marriage in promoting HBI prevention practices.

Second-year nursing students demonstrated a higher likelihood of poor HBI prevention practice compared to their third-year counterparts. An approach employed in Ghana by Osei et al. [27] differed from ours. This is due to the comparison made between first-year and second-year students in this study, whereas our study compares students between the second and third years. However, prolonged education and training provide individuals with the necessary skills and knowledge to effectively implement preventative measures, like the good practice of HBI [23].

The current study found that nursing students who have a family history of HBI exhibit a decreased likelihood of engaging in inadequate HBI prevention practices, in contrast to those who lack such a history. Ahmad et al. [40] and Brouard et al. [44] found that people with a family history of HB tend to have better knowledge about the disease than those without such a familiar background. Furthermore, knowledge of one’s family health history can provide valuable information regarding their predisposition to specific illnesses or diseases, allowing for informed decisions regarding lifestyle choices and preventative practices [45] and this information can facilitate the navigation of the healthcare system and result in the provision of optimal medical care.

### Strength and limitation

This is one of the first studies to look into the factors that affect the practice towards the prevention of HBI among nursing students in the southern region of Bangladesh. Thus, the study’s findings give baseline data that will aid policymakers and public health specialists in developing preventive strategies to reduce HBI in Bangladesh. Another significant aspect of this study is its large sample size, as well as its appropriate methodological and statistical procedures. However, some limitations of our study should be considered. For example, we cannot determine the causal associations due to the cross-sectional study design. During the pre-testing phase of the questionnaire a small group of students were involved, and the same school was used for data collection. It is understandable that some questions may have been discussed by these students with their classmates who participated in the study later. Since the data was collected by the self-administered questionnaire, self-reported bias may be present. Furthermore, some environmental impacts may influence the students during data collection. Such as, the classroom used for data collection was designed for examinations which may put pressure on the students as they are attending the real exam. Despite all these probable influences, it is noteworthy that the data collection went smoothly without any external pressure. The participants completed the questionnaire independently. A clear clarification was given to all the students that their participation was entirely voluntary and that it was not an examination.

## Conclusion

An undesirable level of KAP scores related to the prevention of HBI among nursing students was found in our study. We observed that factors such as low knowledge, suboptimal attitude and other variables like age, marital status, academic status and family history with HBI play a role in shaping the practice of HBI prevention. Specifically, we proposed some recommendations to effectively enhance the prevention practice of HBI and promote the overall health of nursing students. Firstly, in health services, hepatitis B screening, treatment and vaccination services must be free or subsidized. It will ensure that all necessary health care resources have easy access to nursing students when they are practicing. Secondly, the establishment of advocacy and institutional policies. These institution-based policies have to be established for the prevention of HBI. Advocacy efforts can help increase commitment to this cause and raise awareness, which helps to enhance the tendency of adequate practice among students. Thirdly, education and awareness. An environment has to be created through education and awareness programs dedicated to the prevention practice of HBI. These programs should provide knowledge and skills of practice to the nursing students, which they need to protect themselves and others. Finally, skill development. Development of potential skills related to the prevention of HBI, such as strategies to avoid needle stick injuries. Most importantly, professional ethics in health care settings must be emphasized to ensure good practice.

### Electronic supplementary material

Below is the link to the electronic supplementary material.

Supplementary Material 1

## Data Availability

The datasets used and/or analyzed during the current study are available from the corresponding author on reasonable request.
