# Supplementary material for: Assessment of preventive practices towards hepatitis B infection among nursing students in Bangladesh: role of knowledge, attitudes and sociodemographic factors
Source: BMC Nurs. 2024 Mar 21;23:190. doi: 10.1186/s12912-024-01870-8 (PMC10956232; doi:10.1186/s12912-024-01870-8)
Supplement: Supplementary file 1 — Supplementary Material 1 [file 12912_2024_1870_MOESM1_ESM.docx]

**Supplementary Table 1:** Multinomial logistic regression analysis (unadjusted model) for determining factors affecting practice of hepatitis B infection prevention (n = 737)

| Variables |  | Practice of hepatitis B infection prevention | | | | | |
| --- | --- | --- | --- | --- | --- | --- | --- |
|  |  | Moderate | | | Poor | | |
|  |  | OR | 95% CI | p-value | OR | 95% CI | p-value |
| Knowledge | High | Ref. |  |  | Ref. |  |  |
|  | Moderate | 2.431 | 1.35-4.39 | **0.003** | 4.003 | 2.38-6.73 | **0.000** |
|  | Low | 2.567 | 1.299-5.07 | **0.007** | 2.674 | 1.45-4.93 | **0.002** |
| Attitude | Good | Ref. |  |  | Ref. |  |  |
|  | Moderate | 5.393 | 2.76-10.54 | **0.000** | 6.115 | 3.45-10.85 | **0.000** |
|  | Poor | 4.146 | 2.17-7.93 | **0.000** | 5.863 | 3.39-10.12 | **0.000** |
| Age | 19-20 | 1.207 | 0.30-1.32 | 0.222 | 1.207 | 0.65-2.24 | 0.553 |
|  | 21-22 | 0.717 | 0.43-1.197 | 0.203 | 0.772 | 0.487-1.22 | 0.270 |
|  | 23-24 | Ref. |  |  | Ref. |  |  |
| Gender | Male | 1.152 | .579-2.293 | .686 | 1.286 | .708-2.336 | .409 |
|  | Female | Ref. |  |  | Ref. |  |  |
| Religion | Hinduism | 1.234 | .784-1.943 | .364 | 1.325 | .892-1.969 | .163 |
|  | Muslim | Ref. |  |  | Ref. |  |  |
| Marital Status | Married | 0.186 | 0.05-0.67 | **0.010** | 0.357 | 0.16-0.80 | **0.012** |
|  | Unmarried | Ref. |  |  | Ref. |  |  |
| Academic Status | 2^nd^ year | 1.190 | 0.75-1.88 | 0.460 | 1.520 | 1.02-2.26 | **0.040** |
|  | 3^rd^ year | Ref. |  |  | Ref. |  |  |
| Monthly Family Income | ≤ 20,000 BDT | 1.085 | .475-2.478 | .846 | 1.128 | .551-2.306 | .742 |
|  | 21,000 - 40,000 BDT | 1.038 | .425-2.536 | .934 | 1.011 | .466-2.195 | .978 |
|  | >40,000 BDT | Ref. |  |  | Ref. |  |  |
| Educational Qualification of Father | Not Educated or primary level | .933 | .466-1.871 | .846 | .942 | .505-1.758 | .852 |
|  | Secondary or Higher Secondary level | .721 | .387-1.343 | .303 | .893 | .513-1.552 | .687 |
|  | Graduated or above | Ref. |  |  | Ref. |  |  |
| Educational Qualification of Mother | Not Educated or primary level | 1.832 | .699-4.806 | .218 | 2.099 | .947-4.651 | .068 |
|  | Secondary or Higher Secondary level | 2.015 | .804-5.053 | .135 | 1.881 | .881-4.017 | .103 |
|  | Graduated or above | Ref. |  |  | Ref. |  |  |
| Family History with HB Infection | Yes | 0.357 | 0.15-0.84 | **0.019** | 0.143 | 0.06-0.35 | **0.000** |
|  | No | Ref. |  |  | Ref. |  |  |
| Note: Bolded values indicate statistically significant (p<0.05). | | | | | | | |
